# Ferroptosis-mediated osteoclast-osteoblast crosstalk: signaling pathways governing bone remodeling in osteoporosis

**DOI:** 10.1186/s13018-025-06315-9

**Published:** 2025-10-13

**Authors:** DuJiang Yang, GaoWen Gong, JiaFeng Song, JunJie Chen, Shuang Wang, Jingchi Li, GuoYou Wang

**Affiliations:** The Affiliated Traditional Chinese Medicine Hospital of Southwest Medical University, Luzhou, China

**Keywords:** Ferroptosis, Osteoporosis, GPX4, Bone metabolism, Signalling pathway, Lipid peroxidation, TFR1

## Abstract

**Background:**

Osteoporosis is a metabolic bone disease characterized by disruption of bone homeostasis, resulting from an imbalance between osteoblast-mediated bone formation and osteoclast-driven bone resorption. Emerging evidence implicates ferroptosis, an iron-dependent form of regulated cell death driven by lipid peroxidation, in this process. Key molecular hallmarks include glutathione peroxidase 4 (GPX4) inactivation and the intracellular generation of unstable hydroxyl radicals and reactive oxygen species (ROS) accumulation. These accumulated ROS further participate in the oxidation of polyunsaturated fatty acids (PUFAs), generating and accumulating lipid peroxides that ultimately compromise cellular membranes and trigger ferroptosis. This review explores the regulatory mechanisms of ferroptosis, elucidating the roles of key signaling molecules, cytokines, and pathways involved in osteoporosis pathogenesis.

**Methods and materials:**

This review synthesizes recent advances, delineating core pathways(e.g., Xc-GSH-GPX4 axis, FSP1-CoQ10-NADPH, and DHODH-CoQ10 and key molecules (TFR1, FPN, ALOX15) regulating osteoporosis. We emphasize that ferroptosis dysregulation in osteoblasts and osteoclasts disrupts cellular redox balance, impairing bone microstructure and biomechanical strength, thereby accelerating osteoporosis progression. Mechanistically, iron overload promotes ROS production via the Fenton reaction, inactivates GPX4, and triggers a lipid peroxidation cascade. Targeting ferroptosis represents a novel governing strategy to restore bone homeostasis. We discuss unresolved questions regarding ferroptosis regulation in clinical osteoporosis treatment and outline future prospects involving molecular activators, precision delivery systems, and integration of cutting-edge technologies, providing a theoretical foundation for basic research and clinical applications.

## Introduction

### Osteoporosis definition and background

Osteoporosis is a systemic metabolic disorder characterized by reduced bone mineral density (BMD), diminished bone strength, increased fragility, microarchitectural deterioration, trabecular thinning, and elevated fracture risk [1]. The condition and its related fractures impose a substantial socioeconomic burden on individuals, families, and society. It is projected that by 2050, China alone will have an osteoporotic population of 212 million [2, 3]. Numerous risk factors contribute to osteoporosis, including gender, age, genetic predisposition, reproductive status, inadequate calcium intake, and physical inactivity. Under various physicochemical conditions or cellular stressors, the differentiation of bone marrow mesenchymal stem cells (BMSCs) into osteoblasts is markedly impaired [4]. Although associated with microcirculatory impairments and metabolic dysregulation, the core pathophysiology of osteoporosis (OP) fundamentally stems from a disruption in bone homeostasis [5].Fracture risk assessment in OP typically relies on radiographic techniques such as X-rays or BMD measurements. According to the World Health Organization, osteoporosis is diagnosed when a patient’s T-score (BMD) is 2.5 standard deviations or more below the mean for a young healthy reference population [6]. OP is also closely linked to hormonal changes, advanced age, radiotherapy, glucocorticoid therapy, and is highly prevalent among postmenopausal women and the elderly [3].As an essential trace element, iron plays crucial biological roles. Accumulating evidence indicates that ferroptosis acts as a key mechanistic driver in osteoporosis, disrupting cellular redox balance, impairing bone remodeling, and adversely affecting bone mass, microarchitecture, and biomechanical properties—ultimately leading to bone loss and fractures [7]. Furthermore, ferroptosis promotes degradation of the bone matrix, particularly type I collagen, and triggers pronounced inflammatory responses, contributing to overall bone mass reduction and disease progression [8].

### Overview of bone metabolic processes

Bone is a highly dynamic mineralized connective tissue characterized by a porous structure comprising blood vessels, various cell types, and hydroxyapatite crystals. By weight, it primarily consists of minerals (50–70%), organic matrix (20–40%), water (5–10%), and lipids (< 3%) [9], with exact proportions varying according to bone type and anatomical region. The process of bone remodeling occurs through five sequential phases. Activation: This phase is initiated by local mechanical or hormonal signals detected by osteoblasts [10]. Resorption: Mature osteoclasts secrete matrix metalloproteinases (MMPs) and other enzymes to degrade both mineral and organic components of the bone matrix, resulting in the formation of resorption pits known as Howship’s lacunae. The resorbed bone is subsequently replaced by newly formed osteoid [11]. Reversal: Osteoclasts undergo apoptosis upon completion of resorption. Signaling molecules, such as transforming growth factors, released during the resorption process recruit osteoblasts to the resorption sites to initiate new bone formation and mineralization [11]. Formation: Osteoblasts synthesize and deposit an organic matrix rich in type I collagen. Subsequent mineralization leads to the deposition of hydroxyapatite crystals, during which water is gradually expelled from the matrix [12]. Termination: The cycles of bone resorption, matrix formation, and mineralization are concluded. In healthy adult bone, these processes are tightly balanced to maintain skeletal integrity and homeostasis [12].

### Current status of ferroptosis research

#### Definition of ferroptosis

In 2003, Dolma et al. identified erastin, a compound that exhibits selective lethality toward RAS-mutant cancer cells by triggering a unique form of cell death distinct from apoptosis. This process was characterized by the absence of nuclear condensation, DNA fragmentation, caspase activation, and insensitivity to caspase inhibitors [13–15]. In 2012, Dixon et al. formally defined this mechanism as “ferroptosis”—an iron-dependent, non-apoptotic mode of cell death that is morphologically, biochemically, and genetically unique [16]. Morphological hallmarks of ferroptosis include cell rounding with condensed but intact plasma membranes, preserved nuclear architecture without chromatin condensation, and distinctive mitochondrial alterations such as organelle shrinkage, increased membrane density, reduced volume, and loss of cristae [17, 18]. At the molecular level, ferroptosis is driven primarily by inactivation of glutathione peroxidase 4 (GPX4) and the iron-dependent accumulation of unstable hydroxyl radicals and reactive oxygen species (ROS) through the Haber–Weiss and Fenton reactions [16, 19]. These events lead to impaired cystine uptake, depletion of glutathione (GSH), inhibition of the cystine/glutamate antiporter (System Xc⁻), and aberrant accumulation of ferric ions and ROS [20]. The resulting ROS promote lipid peroxidation of polyunsaturated fatty acids (PPUFAs), generating cytotoxic lipid peroxides (such as conjugated dienes and 4-hydroxynonenal) that enhance membrane fragility and ultimately execute ferroptotic cell death. The Fenton reaction, in which free iron catalyzes the conversion of hydrogen peroxide (H₂O₂) into highly reactive hydroxyl radicals (HO·), plays a central role [21]. Genetically, ferroptosis is regulated by genes involved in iron metabolism and storage, including SLC11A2, TFRC, and FTL [22].

Ferroptosis can be initiated through either transporter-dependent (exogenous) or enzyme-regulated (endogenous) pathways. Iron-containing lipoxygenases (LOXs) act as major promoters of ferroptosis by generating lipid hydroperoxides in a process that requires acyl-CoA synthetase long-chain family member 4 (ACSL4)-mediated lipid biosynthesis [23]. Conversely, GPX4 serves as a central inhibitor of ferroptosis; its antioxidant activity depends on GSH, which is synthesized from cysteine imported via the solute carrier family 7 member 11 (SLC7A11) transporter [24]. Excessive ROS accumulation leads to peroxidation of PUFAs within cellular and organellar membranes, mitochondrial dysfunction, dysregulated protein synthesis, and DNA damage, collectively culminating in ferroptotic cell death [25]. Conditions that predispose cells to ferroptosis include impaired GSH synthesis, inactivation of GPX4, reduced cysteine uptake, elevation of labile iron pools (LIPs), and accumulation of Fe^2^⁺, all of which facilitate ROS-driven chain reactions of PUFA peroxidation.

#### Oxidative stress microenvironment in osteoporosis drives ferroptosis

In the pathological progression of osteoporosis, the oxidative stress microenvironment initiates and amplifies ferroptosis through multifaceted mechanisms, establishing a vicious cycle of “oxidative stress–ferroptosis–cell death”. Firstly, oxidative stress exacerbates dysregulation of iron metabolism. Within the bone microenvironment, persistently elevated reactive oxygen species (ROS) activate the NF-κB signaling pathway, leading to upregulation of transferrin receptor 1 (TFR1) and enhanced iron uptake in osteoblasts and osteoclast precursors. Concurrently, ROS oxidize thiol groups on ferroportin (FPN), impairing its stability and inhibiting iron export. Secondly, oxidative stress and ferroptosis form a self-amplifying loop. ROS-driven lipid peroxidation—potentiated by the Fenton reaction, wherein Fe^2^⁺ catalyzes H₂O₂ into highly reactive hydroxyl radicals—directly attacks polyunsaturated fatty acids (PUFAs) within membrane phospholipids, generating lipid hydroperoxides (PUFA-OOH). This peroxidative cascade disrupts plasma membrane integrity, collapses mitochondrial membrane potential, and damages cristae structure, further amplifying ROS generation. Ultimately, these alterations cause irreversible plasma membrane rupture and mitochondrial disintegration, culminating in ferroptotic cell death. Moreover, the oxidative stress microenvironment serves as a central driver of ferroptosis in osteoporosis by reprogramming iron metabolism, compromising antioxidant defenses, accelerating lipid peroxidation, and modulating cellular differentiation responses. This mechanism not only directly promotes osteoblast death and bone matrix degradation but also exacerbates systemic metabolic dysregulation through osteoclast activation and remodeling of the bone marrow niche. Therefore, this review focuses on elucidating the mechanisms through which ferroptosis, under oxidative stress, acts as a critical molecular bridge linking pathological microenvironmental cues to aberrant bone remodeling in osteoporosis.

#### Cellular iron cycle and regulation

Iron is an essential trace element for eukaryotic cellular function, obtained via two primary routes: direct dietary absorption and indirect acquisition through macrophage-mediated phagocytosis and autophagy of senescent erythrocytes and organelles [26, 27]. In mammalian cells, iron is acquired primarily via transferrin receptor (TFR)-mediated endocytosis. Dysregulated iron metabolism is a hallmark of ferroptosis. Circulating Fe^3^⁺ binds to transferrin (Tf), forming a Tf–Fe complex that delivers iron to tissues and cells [28]. Cells with high iron demand express abundant transferrin receptor 1 (TFR1), which binds Tf–Fe and internalizes it through clathrin-dependent endocytosis [29]. The internalized TFR1–Tf–Fe complex traffics to endosomes, where the metalloreductase STEAP3 reduces Fe^3^⁺ to Fe^2^⁺ [30]. This Fe^2^⁺ is then transported into the cytosol by ZIP8/14 or DMT1 [30]. Intracellular iron is either directed to the labile iron pool (LIP) for immediate use or stored in ferritin. Iron can be mobilized from ferritin and delivered to mitochondria as needed. Excess Fe^2^⁺ is exported via ferroportin (FPN1) and bound extracellularly by ferritin heavy chain 1 (FTH1) and light chain 1 (FTL1) [31].

#### The process of ferroptosis

Ferroptosis involves several critical regulatory nodes.First, transferrin receptor 1 (TFR1) modulates the influx rate and quantity of Fe^2^⁺ into cells, thereby exerting a protective effect against ferroptosis and subsequent lipid peroxidation [13]. Second, the cystine/glutamate antiporter system Xc⁻, located on the plasma membrane, plays an essential role in maintaining intracellular levels of glutathione (GSH) and oxidized glutathione (GSSG), which are critical intracellular antioxidants [32]. Inhibition of the SLC7A11 subunit of system Xc⁻ can induce cytosolic ferroptosis. Third, glutathione peroxidase 4 (GPX4) facilitates the synthesis of GSH and GSSG [33]. More importantly, GPX4 utilizes its catalytic activity to reduce phospholipid hydroperoxides into non-toxic phosphatidyl alcohols, thereby preserving lipid bilayer homeostasis and preventing ferroptosis [15].

**Fig. 1:**
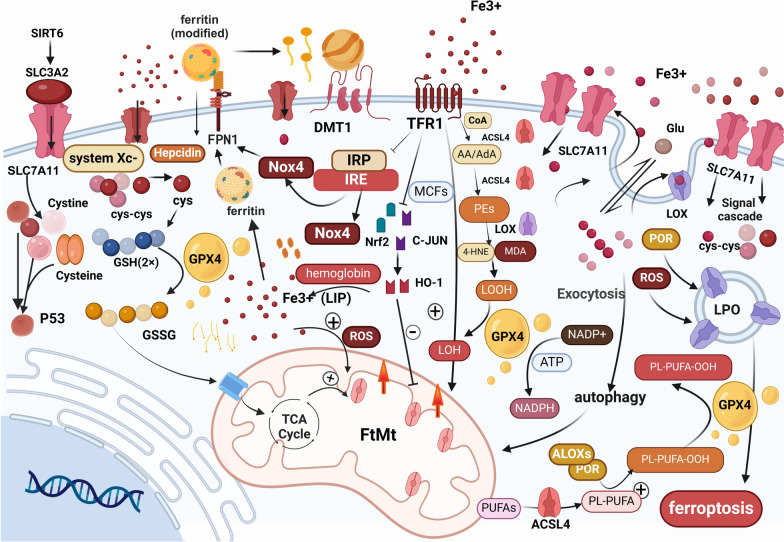
This figure illustrates the dynamic process of ferroptosis, the core molecular regulatory pathways, the key signalling pathway regulatory networks and upstream and downstream influencing factors, as well as the operational mechanisms of different transporters. Specifically, it includes the roles of various molecules such as the Xc-GSH-GPX4 axis, TCA cycle, NADPH, PUFA-PL–OOH, LPO, and ROS through different pathways in the ferroptosis process

Fourth, the collaboration between ferritin heavy chain (FTH) and nuclear receptor coactivator 4 (NCOA4) mediates ferritinophagy—the autophagic degradation of ferritin—resulting in the release of substantial amounts of iron ions and ultimately promoting ferroptosis [34]. Furthermore, ferroptosis suppressor protein 1 (FSP1) inhibits ferroptosis in both the cytosol and mitochondria by reducing lipid peroxidation, thereby preserving the structural integrity of cellular membranes [22] (Fig. 1 and Table 1).

**Table 1.** The dynamic process of iron death, key molecular events, and pathological manifestations in osteoporosis

Stage	Core biological processes	Key molecular events	Pathological manifestations in osteoporosis
Triggering phase	Iron overload formation	TFR1↑: Overexpression of transferrin receptor → Increased iron uptake	Bone marrow iron deposition, serum ferritin ↑
		FPN↓: Inhibition of iron export protein → Intracellular iron accumulation	
		NCOA4-mediated iron autophagy: Ferritin degradation → Increased release of free Fe^2^⁺	
	Lipid substrate preparation	ACSL4↑: Activation of AA/AdA to generate PUFA-CoA	Increased PUFA content in osteocyte membranes
		LPCAT3↑: Integration of PUFA into membrane phospholipids (PE)	
Execution phase	Reactive oxygen species burst	Fenton reaction: Fe^2^⁺ + H₂O₂ → ·OH	Mitochondrial membrane potential↓
		Mitochondrial ETC dysfunction: Complex I/III electron leakage → O₂·⁻↑	
	Lipid peroxidation cascade	Enzymatic pathway: ALOX15 oxidises PUFA-PL → PL-OOH	-
		Non-enzymatic pathway: ·OH attacks PUFA double bonds → lipid radical chain reaction	
Defence failure phase	Antioxidant system collapse	SLC7A11↓: Cysteine uptake impaired → GSH synthesis↓	Bone tissue GSH/GSSG ratio↓ CoQ10 levels↓
		GPX4 inactivation: PL-OOH cannot be reduced → Toxicity accumulation	
		FSP1-CoQ10 axis inhibition: Lipid radical scavenging failure	
Terminal phase	Cell membrane rupture	PL–OOH disrupts membrane integrity → Ion imbalance	Bone cell LDH release ↑
	Effects on the bone microenvironment	Osteoblasts: ↓ mineralisation nodule formation, ↓ Osterix/Runx2 expression	Trabecular number/thickness, ↓ bone biomechanical strength
		Osteoclasts: ↑ TRAP activity, ↑ bone resorption pit area	
		MSCs: ↑ adipogenic differentiation, ↓ osteogenic differentiation	
References	[22, 27, 30, 35–37]	[30, 31, 38–44]	[19, 27, 36, 39, 45, 46]

#### Regulation of ferroptosis by GPX4

GPX4 is a selenium-dependent glutathione peroxidase that requires glutathione as a cofactor. It consists of 170 amino acids with a theoretical molecular weight of approximately 19 kDa [45]. The GPX4 gene contains seven exons. Among the glutathione peroxidase family (GPX1–GPX8), GPX4 is unique in its ability to efficiently reduce lipid hydroperoxides and protect cells from oxidative damage [16]. Specifically, GPX4 catalyzes the reduction of phospholipid hydroperoxides (PUFA-PL-OOH) to their corresponding phosphatidyl alcohols, thereby inhibiting the propagation of lipid peroxidation and preventing ferroptosis [47]. Lipid hydroperoxides are either degraded by GPX4 or reduced to non-toxic alcohols, concurrently oxidizing glutathione (GSH) to glutathione disulfide (GSSG). Glutathione reductase then regenerates GSH from GSSG using NADPH as a reducing agent [33, 38]. Cysteine, imported via the system Xc⁻ cystine/glutamate antiporter, is reduced to cysteine and serves as a key substrate for GSH synthesis [48]. Consequently, impaired GPX4 function results in the accumulation of toxic lipid peroxides, triggering ferroptosis and promoting osteoclast-mediated degradation of the bone matrix [49].

#### Indirect regulation of ferroptosis by GSH

GSH synthesis depends on Gln-derived Glu and cystine. Cystine entering the cell is reduced to cysteine by reductase. Glutamate cysteine ligase condenses glutamate with cysteine in an ATP-dependent manner to generate γ-glutamylcysteine. Glutathione synthase then condenses this with glycine to produce GSH [50, 51]. The rate-limiting enzyme for GSH production, γ-glutamylcysteine ligase (GCL), is critical for normal cellular function [39]. Under unfavorable conditions, inhibition of the glutamine transporter SLC38A1/ASCT2 enhances Gln catabolism and accelerates ferroptosis [52].

#### Indirect regulation of ferroptosis by NrF2

Nuclear factor erythroid 2-related factor 2 (Nrf2), a member of the CNC-bZIP family, serves as a central transcriptional regulator of antioxidant responses and plays a critical role in bone development and metabolism [40]. It contains seven conserved Neh domains that modulate its transcriptional activity. Under basal conditions, Kelch-like ECH-associated protein 1 (Keap1) targets Nrf2 for ubiquitination and proteasomal degradation in the cytoplasm [53]. Under oxidative stress, Keap1 undergoes conformational changes, leading to Nrf2 stabilization and release. Subsequently, Nrf2 translocates into the nucleus, forms heterodimers with small Maf (sMaf) proteins, and binds to antioxidant response elements (AREs), thereby initiating the transcription of cytoprotective genes [54]. Nrf2 directly upregulates the expression of GPX4 [55] and indirectly enhances the production of heme oxygenase-1 (HO-1), NADPH, and NADH [38]. Through these mechanisms, Nrf2 supports osteoblast survival and function and suppresses osteoclast activation [41].

#### Regulation of ferroptosis by heat shock protein A5 and capsaicin receptor

By directly binding to and favorably regulating the expression of GPX4 protein, heat shock protein (HSP) A5 inhibits the osteogenic response and ferroptosis in osteoblasts, slowing the progression of bone defects [56]. Ferroptosis signature genes were shown to be preferentially expressed in ferroptosis osteoblast populations, and capsaicin receptor (TRPV1) was screened for potential targets for anti-ferroptosis both in vivo and in vitro using single-cell RNA sequencing analysis of human chondrocyte [57]. TRPV1 avoids ferroptosis by upregulating GPX4 expression. TRPV1 prevents ferroptosis by upregulating GPX4 expression [58]. Similarly, the upregulation of GPX4 expression upon activation of TRPV1 in osteoblasts prevents osteoblastic ferroptosis.

#### Indirect regulation of ferroptosis by ALOX15

In the context of cancer and inflammation, PUFAs, linoleic acid (LA), arachidonic acid (AA), and the enzymes that metabolize these PUFAs have come to light as significant regulators of osteogenesis [42]. AA is a 20-carbon fatty acid with phospholipase A2 activity [25]. Lipid peroxidation during ferroptosis is impacted by lipoxygenases and cyclooxygenases, which further stimulate lipid metabolism in response to the release of AA from the nuclear membrane’s phospholipids [25].

#### Potential regulatory role of FSP1 on ferroptosis cellular

The FSP1-Coenzyme Q10-NAD(P)H pathway exists as an independent parallel system, which, in synergy with GPX4 and GSH, can inhibit phospholipid peroxidation and ferroptosis [22, 23]. FSP1 is a key component of the nonmitochondrial Coenzyme Q antioxidant system, and as an upstream protein of CoQ10, FSP1 agonises NADPH and catalyses the regeneration of CoQ10 [53]. FSP1 acts as an upstream protein of CoQ10, agonising NADPH and catalysing the regeneration of CoQ10, a system that acts in parallel with the typical GSH-based GPX4 pathway [59]. FSP1 is localised in the cell membrane, and as a NAD(P)H-dependent oxidoreductase, it can reduce CoQ10 and capture lipid peroxidation radicals, thus inhibiting lipid peroxidation and ferroptosis [53].

### Important molecules associated with the process of ferroptosis

#### SLC7A11

The cystine/glutamate antiporter (System Xc⁻) is responsible for cystine reduction to cysteine and antioxidant GSH synthesis [60]. System Xc⁻ functions as a glutamate/cystine antiporter, importing one cystine molecule in exchange for one intracellular glutamate molecule. It is a sodium-independent antiporter composed of two subunits linked by a disulfide bond: the light chain subunit SLC7A11 and the heavy chain subunit SLC3A2 (CD98) [61–63]. SLC7A11 mediates glutamate/cystine exchange activity, while SLC3A2 stabilizes SLC7A11 [63]. SLC11A2 allows only divalent iron ions to pass; Fe^3^⁺ is reduced to Fe^2^⁺ by STEAP3 in an acidic environment and transported to the cytoplasm by DMT1 [62]. Extracellular cystine is imported via SLC7A11, and GSH is synthesized from cysteine via NADPH-dependent reduction [64]. γ-GCS catalyzes the binding of cysteine to glutamate to form γ-glutamylcysteine. Glutathione synthase then adds glycine to produce GSH. GPX4 uses GSH to reduce lipid hydroperoxides to alcohols, oxidizing GSH to GSSG, which is reduced back to GSH by NADPH consumption [65].

#### ZIP14 (SLC39A14)

Correlation studies of the sequence of Solute Carrier Family 39 Member 14 (ZIP14, also known as SLC39A14) have shown that ZIP14 has nine homologues and is a protein with sequence homology to the estrogen-related gene LIV-1, known as ZIP (ZRT/IRT-related protein) [66]. ZIP14 is mainly located at the plasma membrane and is responsible for the transport of extracellular zinc ions into the cytoplasm [67]. Zip14 has eight transmembrane domains, a short extracellular C-terminus with numerous histidine-rich repeats, and a long extracellular N-terminus.Zip14 can transport non-translocated ions [68]. ZIP14 transports non-transferrin-bound iron and is also regulated by iron. Under conditions of complete transferrin saturation, iron will be present as non-transferrin bound iron (NTBI), thus providing a substrate for ZIP14 [69].

#### TFr1

Transferrin (Tf) is an 80 kDa serum glycoprotein primarily synthesized in the liver, which serves as a key iron-chelating protein and central mediator of systemic iron transport [29]. Tf binds free trivalent iron (Fe^3^⁺) in the blood and delivers it to target cells via a reversible high-affinity interaction [70]. Upon Fe^3^⁺ binding, Tf undergoes a conformational change that facilitates its recognition and binding to transferrin receptor 1 (TfR1) on the cell membrane. The resulting Tf–TfR1 complex is internalized via endocytosis. Within the endosome, Fe^3^⁺ is reduced to ferrous iron (Fe^2^⁺) by the metalloreductase STEAP3 and exported into the cytoplasm through divalent metal transporter 1 (DMT1). Following iron release, the Tf–TfR1 complex is recycled back to the cell surface, where Tf is released. TfR1 is a transmembrane glycoprotein composed of two disulfide-linked subunits of approximately 700 amino acids each, which specifically recognizes and binds Tf through its extracellular domain [28]. This binding triggers clathrin-coated vesicle formation and endocytosis. After iron reduction and dissociation, Fe^2^⁺ is transported via DMT1, and TfR1 returns to the plasma membrane to initiate further rounds of iron uptake [71].

#### FPN

Ferroportin (FPP N) is the sole known cellular iron exporter and is highly expressed in certain cell types critical for systemic iron homeostasis, such as hepatocytes and macrophages. Studies in mice with conditional deletion of FPN in osteoclast lineage cells revealed that FPN deficiency led to mildly elevated intracellular iron levels in osteoclast precursors and concomitant enhancement of osteoclastogenesis, resulting in reduced bone mass [43]. Downregulation of FPN promotes osteoclast differentiation. Increased intracellular free iron stimulates macrophage proliferation and activates the expression of nuclear factor of activated T cells 1 (NFATc1) and PPARG coactivator 1β (PGC-1β) [72, 73]. Furthermore, FPN mutant mice exhibited decreased bone mass and impaired osteogenesis, although osteoclast-related parameters were not significantly altered, supporting the role of FPN as a negative regulator of osteoclast activity [74, 75] **(**Table 2**).**

**Table 2:** The core molecular regulatory pathway of ferroptosis and its physiological role in osteoporosis

Stage	Core biological processes	Key molecular events	Pathological manifestations in osteoporosis
Triggering phase	Iron overload formation	TFR1↑: Overexpression of transferrin receptor → Increased iron uptake	Bone marrow iron deposition, serum ferritin ↑
		FPN↓: Inhibition of iron export protein → Intracellular iron accumulation	
		NCOA4-mediated iron autophagy: Ferritin degradation → Increased release of free Fe^2^⁺	
	Lipid substrate preparation	ACSL4↑: Activation of AA/AdA to generate PUFA-CoA	Increased PUFA content in osteocyte membranes
		LPCAT3↑: Integration of PUFA into membrane phospholipids (PE)	
Execution phase	Reactive oxygen species burst	Fenton reaction: Fe^2^⁺ + H₂O₂ → ·OH	Mitochondrial membrane potential↓
		Mitochondrial ETC dysfunction: Complex I/III electron leakage → O₂·⁻↑	
	Lipid peroxidation cascade	Enzymatic pathway: ALOX15 oxidises PUFA-PL → PL–OOH	–
		Non-enzymatic pathway: ·OH attacks PUFA double bonds → lipid radical chain reaction	
Defence failure phase	Antioxidant system collapse	SLC7A11↓: Cysteine uptake impaired → GSH synthesis↓	Bone tissue GSH/GSSG ratio↓ CoQ10 levels↓
		GPX4 inactivation: PL-OOH cannot be reduced → Toxicity accumulation	
		FSP1-CoQ10 axis inhibition: Lipid radical scavenging failure	
Terminal phase	Cell membrane rupture	PL-OOH disrupts membrane integrity → Ion imbalance	Bone cell LDH release ↑
	Effects on the bone microenvironment	Osteoblasts: ↓ mineralisation nodule formation, ↓ Osterix/Runx2 expression	Trabecular number/thickness, ↓ bone biomechanical strength
		Osteoclasts: ↑ TRAP activity, ↑ bone resorption pit area	
		MSCs: ↑ adipogenic differentiation, ↓ osteogenic differentiation	
References	[22, 27, 30, 35–37]	[30, 31, 38–44]	[19, 27, 36, 39, 45, 46]

### Various metabolic processes undergo ferroptosis to regulate osteogenesis

#### Lipid metabolism and ferroptosis

Ferroptosis is centrally driven by iron-dependent dysregulation of lipid peroxidation, closely associated with the incorporation of arachidonic acid (AA) and adrenic acid (AdA) into phospholipids [76]. Intracellular accumulation of lipid peroxides (LPO) results from an imbalance between their production and elimination [77]. Lipid peroxidation occurs via both non-enzymatic (e.g., Fenton reaction) and enzyme-catalyzed pathways [78]. Non-enzymatic initiation involves abstraction of bis-allylic hydrogen atoms from polyunsaturated fatty acids (PUFAs) within phospholipids, generating carbon-centered phospholipid radicals that react with oxygen to form phospholipid peroxyl radicals [14]. These radicals subsequently abstract hydrogen from adjacent PUFAs, yielding phospholipid hydroperoxides (PL-OOH) and propagating further radical formation. Under Fe^2^⁺ catalysis, phospholipid hydroperoxides decompose into alkoxyl radicals (RO∙), initiating autocatalytic chain reactions that amplify peroxidation [78, 79]. AA is activated by acyl-CoA synthetase long-chain family member 4 (ACSL4) to form AA-CoA, which is esterified by lysophosphatidylcholine acyltransferase 3 (LPCAT3) into phosphatidylethanolamine (PE), providing a substrate for peroxidation. PE-containing AA and AdA are particularly susceptible to peroxidation in the presence of Fe^2^⁺ and hydroxyl radicals (HO∙), ultimately triggering ferroptosis [77, 80]. When antioxidant defenses in bone cells fail to counteract radical amplification, lipid peroxidation propagates across membranes, leading to irreversible membrane damage and ferroptotic cell death.

#### Mitochondria and ferroptosis

Mitochondrial apoptotic proteins localize to the outer membrane, oxidative phosphorylation complexes to the inner membrane cristae, and the stroma houses the mitochondrial genome, protein translation system, citric acid cycle, and fatty acid β-oxidation [81]. Mitochondria induce ferroptosis through three mechanisms: Mitochondrial ROS destroy PUFAs in the mitochondrial membrane under oxidative stress, inducing lipid peroxidation and ferroptosis [82]. As ATP-producing organelles, mitochondria inactivate AMPK, promoting ferroptosis [16]. Mitochondria mediate the TCA cycle and homologous reactions, promoting ferroptosis via metabolic regulation [83]. During ferroptosis, mitochondrial morphology changes significantly, including fragmentation, cristae enlargement, and membrane thickening [84]. Mitochondria contain iron homeostasis regulators (e.g., mitochondrial ferritin, HMOX1, transporter proteins) [84, 85]. Inhibition of HMOX1 prevents cytoskeletal protein multiplication in unfavorable environments and accelerates osteocalcin and osteoprotegerin degradation. Overexpression of mitochondrial ferritin (FtMt) reduces iron overload-induced oxidative stress, inhibiting osteoblast ferroptosis, suggesting FtMt as a potential therapeutic target for osteoporosis [86]. The Tf–TFR complex and lysosomal ferritin breakdown are primary iron sources for mitochondria [70]. FtMt facilitates iron transport to mitochondria for iron-sulfur cluster (ISC) and heme-associated protein synthesis [86]. Overexpression of FtMt reverses erastin-induced ferroptosis by storing excess iron and preventing oxidative damage [87]. Mitochondrial enzymes degrade hemoglobin to produce Fe^2^⁺, leading to ROS and OH∙ generation, mitochondrial membrane potential depolarization, permeability pore opening, and oxidative respiratory chain dysfunction, causing abnormal mitochondrial morphology and function, ultimately accelerating ferroptosis [88, 89].

#### P53-mediated ferroptosis

The tumor suppressor p53 regulates fatty acid production and lipid metabolism enzymes [90] and triggers cellular responses (e.g., apoptosis, cell cycle arrest, senescence) under stress signals [14]. p53 contains two N-terminal transactivation domains (TADs), a DNA-binding domain (DBD), a tetramerization domain (TET), and a C-terminal regulatory domain [91]. p53 upregulation significantly decreases SLC7A11 mRNA and protein levels, confirming SLC7A11 as a p53 target [92]. While ROS alone did not induce cell death, p53 induction combined with ROS caused > 90% cell death, reduced to 40% with the ferroptosis inhibitor ferrostatin-1 (Fer-1), indicating p53 induces ferroptosis [91]. Iron chelators increase p53 stability and enhance p53 expression via HIF-1α [93]. p53 also regulates iron sensor expression, increasing ferritin expression post-transcriptionally [94]. Thus, p53 induces ferroptosis by downregulating SLC7A11, a key component of System Xc⁻ [61]. This regulatory mechanism exhibits cell type-specific effects due to metabolic differences: osteoblasts, which rely on robust antioxidant defenses, are compromised by p53-mediated GPX4 suppression; whereas osteoclasts, dependent on mitochondrial respiration, exhibit enhanced bone resorption capacity under p53-driven oxidative stress. This dual dysregulation accelerates osteoporosis progression through concurrent osteoblast dysfunction and osteoclast activation.

#### ROS-mediated ferroptosis

Reactive oxygen species (ROS) are a group of oxygen-containing chemically reactive molecules, including hydroxyl radicals, superoxides, and peroxides [95, 96]. Ferroptosis is facilitated by mitochondrial ROS generation and can be inhibited by mitochondria-targeted antioxidants or enzymes [97]. This process is primarily mediated through the induction of lipid peroxidation, which can likewise be suppressed by mitochondrial-specific antioxidants or enzymatic interventions [35]. Prior to the recognition of mitochondrial ROS as an inducer of ferroptosis, it was established that mitochondrial ROS also promote apoptosis, suggesting potential molecular crosstalk between these two forms of cell death.

### Other processes closely related to ferroptosis

#### Iron autophagy

Iron autophagy is a form of autophagic cell death characterized by iron-dependent lipid peroxidation [36]. It is an upstream regulator in the ferroptosis cascade, promoting ferroptosis by influencing autophagy-related proteins, ferritin, and NCOA4. NCOA4 acts as a cargo receptor for ferritin, mediating its transport to autophagic vesicles and promoting ferritin degradation for iron release [81]. This involves NCOA4–ferritin complex formation and ferritin degradation. During autophagosome formation, ATG5 and ATG12 are activated by ATG7 and ATG10, forming a stable complex with ATG16L [37]. The ubiquitin-binding protein p62 is recruited to the autophagosome membrane by binding LC3. Ferritin binds to the C-terminal domain of NCOA4 via FTH1, forming an NCOA4–ferritin complex. This complex is co-activated by ATG5–ATG12–ATG16L, while LC3-I binds phosphatidylethanolamine and converts to LC3-II, promoting autophagosome membrane expansion. The complex is isolated in the autophagosome [98], which then interacts with lysosomes [99].

### Various signalling pathways further influence osteogenesis by regulating ferroptosis

#### Regulation of ferroptosis by the Xc-GSH-GPX4 axis

The Xc-system, an amino acid reverse transporter with two subunits, SLC3A2 and SLC7A11, is essential for GSH synthesis [62]. Various inhibitors of cystine/glutamate reverse transporter proteins reduce GSH and ultimately accumulate ROS in the form of lipid hydroperoxides leading to ferroptosis [24]. GPX4 oxidizes glutathione to GSSG while concurrently reducing phospholipid peroxides to their corresponding alcohols [33]. This process is inhibited by RAS selective lethal smallmolecule 3 (RSL3) [100]. The GSSG produced is then reduced to GSH by glutathione reductase using reduced coenzyme II (NADPH), forming a cycle.

#### SLC7A11-GPX4 axis regulation of ferroptosis

System Xc⁻ is an amino acid antiporter ubiquitously expressed in plasma membranes, composed of a disulfide-linked heterodimer between SLC7A11 and SLC3A2 [61]. It mediates the 1:1 exchange of extracellular cystine for intracellular glutamate [101]. Imported cystine is reduced to cysteine and utilized in glutathione (GSH) synthesis. Key upstream regulators of SLC7A11 include Nrf2, p53, and HIF-1α [102]. In vitro, astaxanthin inhibits erastin-induced ferroptosis in osteoblasts, attenuates extracellular matrix degradation, and reduces inflammatory responses, thereby decelerating the progression of osteoporosis (OP) [103]. D-Glycopyrrolate decreases susceptibility to ferroptosis in chondrocytes and osteoblasts by alleviating HIF-2α-mediated suppression of SLC7A11 [104]. Elevated extracellular glutamate or pharmacological inhibitors (e.g., sulfasalazine, erastin, sorafenib) promote ferroptosis by impairing cysteine uptake or inactivating GPX4, resulting in uncontrolled lipid peroxidation [105]. Osteoporotic phenotypes under ferroptotic conditions are more closely associated with local iron chelation within bone tissue than systemic iron levels, suggesting that topical therapeutic agents may offer superior efficacy. Even moderate reduction of GPX4 in osteoblasts may enhance chondrocyte sensitivity to oxidative stress, accelerating OP progression through MAPK/NF-κB-mediated degradation of the extracellular matrix [72].

#### Regulation of ferroptosis by the FSP1-CoQ10-NADPH pathway

The reduced form of coenzyme Q10 (CoQ10) and ubiquinol (reduced coenzyme Q10, CoQ10H2) mediate the ferroptosis suppressor protein1 (FSP1) pathway. By directly trapping the lipid radicals that mediate lipid peroxidation, the reduced form of CoQ10 inhibits lipid peroxidation and ferroptosis [16]. FSP1 can catalyse the regeneration of CoQ10 using NAD(P)H.

#### Regulation of ferroptosis by the DHODH-CoQ10H2 pathway

Dihydroorotate dehydrogenase (DHODH) is a local defence system that mediates mitochondrial peroxidative toxicity by compensating for the loss of GPX4 [53, 106]. DHODH acts as a pyrimidine synthase that reduces CoQ10 to CoQ10H2 on the inner mitochondrial membrane, inhibiting ferroptosis. traps oxygen radicals and inhibits ferroptosis. Especially during the acute inactivation of GPX4, the amount of DHODH increases significantly to promote the production of ubiquinol, which neutralises lipid peroxidation and prevents mitochondrial ferroptosis [107]. In order to counterbalance one another, GPX4 and DHODH suppress mitochondrial lipid peroxidation.

#### Potential regulation of ferroptosis by the GCH1-BH4 pathway or squalene-mediated pathways

GTP cyclohydrolase-1 (GCH1) has recently been reported to inhibit ferroptosis via its metabolites dihydrobiOPterin and tetrahydrobiOPterin (BH4) [108]. BH4 oxidatively degrades phospholipids containing two PUFA tails and accelerates the production of coenzyme Q10. Coenzyme Q10 production. BH4 promotes the synthesis of coenzyme Q10 and has an oxidative degradation effect on phospholipids with two PUFA tails [47, 109]. Under conditions of oxidative stress, the build-up of squalene can effectively prevent membrane PUFA damage or enhance fatty acid metabolism to boost cellular antioxidant levels and avoid ferroptosis.

#### Potential regulation of ferroptosis by NCOP4 and related pathways

The deletion of DNA methyltransferase-1 (DNMT-1) decreases the NCOP4-mediated regulation of ferroptosis, which in turn reduces osteoblastic damage. DNMT-1 is involved in DNA methylation [110]. Cellular ferroptosis results from the concurrent rise in intracellular ferrous iron and these indicators that occurs after ferritin degradation. Nevertheless, the precise targets and mechanisms by which NCOP4-associated regulatory proteins influence ferroptosis remain unknown (Table 3).

**Table 3:** Key signalling pathways regulating ferroptosis and upstream and downstream factors

Pathway name	Core regulatory node	Upstream trigger	Downstream effect	Osteoporosis pathology association
Xc⁻-GSH-GPX4 axis	Erastin → SLC7A11↓	*P*53 activation/HIF-1α	InhibitionGSH depletion → GPX4 inactivation → PL-OOH accumulation	Osteoblast death ↑ → Bone formation ↓
FSP 1-Coenzyme Q10-NADPH	FSP1 phosphorylation	Estrogen receptor signalling	CoQ10 regeneration → Lipid free radical scavenging ↑	Reduced pathway activity in postmenopausal OP
Nrf2/HO-1 pathway	Keap1-Nrf2 dissociation	Keap1-Nrf2 dissociation oxidative stress/iron chelators	HO-1↑ → Iron chelation/GPX4↑	Nrf2 agonists improve bone microstructure
*p*53-SLC7A11 axis	P53 binding to xCT promoter	DNA damage/ROS accumulation	XCT transcription ↓ → Cysteine uptake ↓	Abnormal bone density in *p*53 mutant mice
Mitochondrial iron death axis	FtMt/DHODH-CoQ10	Mitochondrial ROS burst	DHODH compensates for GPX4 inactivation → Resists lipid peroxidation	FtMt overexpression rescues erastin-induced bone loss
References	[21, 24, 30, 36, 43, 44, 53, 73]	[38, 39, 48, 84, 93–96]	[20, 31, 37, 46, 59, 64]	[1, 4–7, 44, 111, 112]

### Ferroptosis mediates related cell changes in bone metabolism

#### Inhibition of osteoblast activity and function by ferroptosis

Osteoblasts, differentiated from mesenchymal stem cells, are the main functional cells in bone metabolism, involved in bone repair and reconstruction through secretion of calcified nodules and collagen fibers [113–115]. Excess iron impairs the biological activity of osteoblasts [116]. Iron promotes serum ferritin production and suppresses osteoblast function through ferroxidase activity, contributing to osteoporotic progression. Runx2, a key transcription factor regulating MSC differentiation into osteoblasts, is downregulated under iron overload, thereby inhibiting osteogenic differentiation [117]. Elevated ROS levels inhibit osteoblast differentiation by disrupting Wnt/β-catenin, PI3K/AKT, MAPK, and Hedgehog signaling pathways, leading to reduced osteoblast generation and disrupted bone remodeling balance [46]. Ferroptosis alters osteogenic marker expression, characterized by reduced alkaline phosphatase activity, impaired mineralized nodule formation, and decreased expression of osteoprotegerin and osteocalcin. Excessive iron induces conformational changes in thioredoxin, further inhibiting osteoblast proliferation and promoting ferroptotic cell death.

#### Ferroptosis promotes osteoclast differentiation and activation

Osteoclasts, derived from monocyte-lineage hematopoietic precursor cells, are terminally differentiated multinucleated cells whose differentiation and function are primarily regulated by two key cytokines: macrophage colony-stimulating factor and receptor activator of nuclear factor-κB ligand [114, 118]. Their mononuclear precursors undergo fusion to form large, specialized multinucleated cells [118, 119]. Ferroptosis promotes osteoclast differentiation, activation, and bone resorption through the following mechanisms: (1) Elevated free iron levels enhance mitochondrial iron content via transferrin receptor 1 (TfR1)-mediated complex formation, boosting mitochondrial respiratory capacity and providing energy to support osteoclast differentiation and bone resorption [111]. (2) Ferroptosis increases the expression of RANKL by inducing osteoclast apoptosis and promoting the differentiation of monocyte-macrophages into osteoclasts, thereby enhancing osteoclast activity. (3) Excess iron generates ROS through the Fenton reaction. ROS-induced oxidative stress stimulates osteoclast activation by modulating the activity of key transcription factors within osteoclasts [44, 112].

#### Ferroptosis inhibits bone marrow mesenchymal stem cell activity and function

Bone marrow mesenchymal stem cells are pluripotent stem cells of mesodermal origin with the potential to differentiate into osteoblasts, osteocytes, adipocytes, muscle cells, endothelial cells, and cardiomyocytes [120, 121]. Osteoporosis results from ferroptosis, which lowers bone density by encouraging the apoptosis of bone marrow mesenchymal stem cells. Specific signaling molecules, including bone morphogenetic proteins, transforming growth factor β, fibroblast growth factor, insulin-like growth factor, and platelet-derived growth factor, regulate MSCs during bone regeneration and remodeling [34, 121]. The Each of the aforementioned signaling pathways acts either directly or indirectly to control the expression of downstream related factors like osteocalcin and alkaline phosphatase, ultimately converging on the Runx2, a crucial node in the regulation of osteogenic differentiation [46].

#### Ferroptosis regulation within the bone marrow niche

The bone marrow niche constitutes a spatially structured ecosystem in which cellular heterogeneity and microenvironmental gradients modulate susceptibility to ferroptosis [122]. Paracrine signaling between osteoblasts and osteoclasts fine-tunes iron homeostasis through dynamic intercellular networks. Furthermore, macrophages and their functional polarization—into pro-inflammatory M1 and pro-regenerative M2 phenotypes—govern tissue repair and disease progression in the musculoskeletal microenvironment [123, 124]. This paradigm extends to the skeletal system, where osteoclasts are increasingly recognized as specialized bone-resident macrophages within the mononuclear phagocyte system and represent an integral component of the immunometabolic axis [125, 126]. These observations collectively imply the existence of a sophisticated oxidative stress–inflammatory network within the bone marrow niche. Inflammatory mediators such as TNF-α and IL-1β exacerbate oxidative stress by suppressing GPX4 expression in osteoblasts and upregulating TFR1 in osteoclast precursors via NF-κB activation [127, 128]. Hypoxia stabilizes HIF-1α in osteocytes, promoting FPN degradation and elevating local Fe^2^⁺ pools that drive Fenton chemistry within osteoblasts [129]. This iron stratification underlies the heightened ferroptosis sensitivity of osteoblasts compared to stromal fibroblasts. A metabolic symbiosis exists: osteoclast-derived hepcidin inhibits iron export from osteoblasts by internalizing FPN, thereby sequestering iron within the bone matrix; conversely, osteoblast-secreted cystine precursors protect osteoclasts from erastin-induced ferroptosis via xCT-dependent glutathione synthesis [130, 131]. Ferroptosis is increasingly redefined as a tissue-level redox relay mechanism rather than an autonomous cell death process, encompassing: (i) Vesicular transfer of ACSL4 from macrophages to osteoblasts, priming phospholipid peroxidation; (ii) Intercellular mitochondrial trafficking from mesenchymal stem cells (MSCs) to osteoclasts, wherein FSP1–CoQ10 complexes attenuate lipid peroxidation via nanotubular transport; (iii) Mechanosensitive regulation of SLC7A11 by osteocytic dendritic processes across the lacunar-canalicular system, modulating redox capacity in response to mechanical stimuli [132, 133]. Targeting niche-specific regulators—such as myeloid HIF-2α to disrupt spatial iron accumulation—may facilitate spatially confined anti-ferroptosis therapies, offering a more precise alternative to systemic antioxidant strategies (Table 4).

**Table 4:** Key molecules differences in sensitivity to ferroptosis and differences in effects among different bone cells

Cell type	Osteoblasts	Osteoclasts	Bone marrow MSCs	Osteocytes
Ferroptosis sensitivity	High	Stage-dependent sensitivity	Moderate	Low
Functional consequences	GPX4↓, ACSL4↑, FtMt↓ → Lipid ROS↑	Precursor cells: TFR1↑ → Iron accumulation → Differentiation↑	Iron overload → Runx2↓ → Osteogenic differentiation arrest	Sclerostin↑ → Wnt pathway inhibition → Increased resistance to iron-induced cell death(Not mentioned)
		Mature cells: FPN↑ → Iron export → Survival↑	Lipid peroxidation → PPARγ↑ → Adipogenic differentiation↑	
Key molecular events	ALP activity↓, mineralisation nodules↓, collagen secretion↓	Bone resorption area↑, TRAP activity↑	Reduced osteoblast pool → Bone formation↓	Mechanical signal transduction impairment → Bone remodelling dysfunction
Therapeutic intervention strategies	GPX4 activators/iron chelators	TFR1 inhibitors (early intervention)	Nrf2 activator/Runx2 expression enhancement	Sclerostin antibody
References	[4, 11, 15, 66, 117]	[30, 31, 38, 40, 43]	[19, 27, 42, 44, 48]	[4]

## Summary and perspective

Ferroptosis research is advancing from phenomenological observation toward mechanism-driven investigation. Future studies should focus on spatial regulation (subcellular localization), temporal intervention (disease-stage specificity), and immune-metabolic crosstalk to establish a multi-dimensional therapeutic framework. Ultimately, integrating nanomaterials, physical modulation, and gene editing into clinical translation pipelines will enable precise ferroptosis-targeted therapies for osteoporosis and related disorders. Specific strategic directions include:

1. Development of GPX4 activators: GPX4 inactivation drives lipid peroxidation accumulation [24]. Novel small molecules or gene therapies restoring GPX4 activity could enhance osteoblast antioxidant capacity and block the lipid peroxidation cascade.
2. Mapping organelle interactions: Mitochondria drive ferroptosis via metabolic reprogramming (TCA cycle), ion flux, and membrane lipid peroxidation [78]. Combining cryo-electron microscopy and spatial omics can elucidate molecular bridges between mitochondrial cristae remodeling and lipid peroxidation diffusion, mapping the ‘ferroptosis organelle interactome’.
3. Development of SIRT3 agonists: SIRT3 regulates mitochondrial deacetylation. Its activators could restore mitochondrial membrane potential, inhibit BNIP3-mediated pathological mitophagy, and break the ferroptosis-mitochondrial damage vicious cycle [134, 135].
4. Development of membrane-localized targeted delivery systems: FSP1 localizes to membranes via N-myristoylation [22]. Designing myristoylation analogues or ‘artificial myristoylation’ nanocarriers could stabilize FSP1 and achieve precise subcellular drug localization (e.g., mitochondrial membrane) [135].
5. Multi-omics prediction and gene-drug network screening: Integrating single-cell multi-omics (scRNA-seq, lipidomics, metabolomics) and constructing ‘ferroptosis gene-drug’ interaction networks based on HIFU-transcriptome data could identify synergistic ferroptosis-inducing drug combinations [136, 137].

The application of ferroptosis modulation in osteoporosis treatment transcends the conventional “calcium–vitamin D” paradigm, shifting toward metabolic intervention, epigenetic regulation, and precision targeting. Future research should prioritize developing molecular activators and combining advanced technologies—such as gene editing and single-cell spatial omics—with clinical resources (e.g., osteoporosis biobanks). Ferroptosis-targeted therapy represents a promising avenue for personalized medicine and may fundamentally transform osteoporosis management.

## Data Availability

No datasets were generated or analysed during the current study.

## Abbreviations

OP: Osteoporosis
BMD: Bone mineral density
OC: Osteoclasts
OB: Osteoblasts
GPX4: Glutathione peroxidase4
MMPs: Matrix metalloproteinases
BMP: Bone morphogenetic protein-2
SLC11A2: Solute carrier family 11
TFRC: Transferrin rreceptor
PUFA: Polyunsaturated fatty aacid
STEAP: Six-segment transmembrane epithelial antigen of the prostate
FTL1: Ferritin light chain 1
BMSCs: Bone marrow mesenchymal stem cells
TRPV1: Transient receptor potential vanilloid-1
mTOR: Mammalian target of rapamycin
GSH: Glutathione
GSSG: Oxidized glutathione
Nrf2: Rbc-derived related factor 2
IL-: Interleukin-
AMPK: AMP-activated protein kinase
ATG: Autophagy-related gene
NF-κB: Nuclear factor kappa-B
AA: Arachidonic acid
LA: Linoleic acid
LC3: Microtubule—associated protein light chain 3
BCL2: B-cell lymphoma-2
TLR2: Toll-like receptors-2
PI-3K: Phosphatidylinositol 3-kinase
ULK1: Unc-51 like kinase 1 gene
ATP: Adenosinetriphosphate
ROS: Reactive oxygen species
BINP3: BCL2 interacting protein 3
TNF: Tumor necrosis factor
MAPK: Mitogen-activated protein kinase
RANKL: Receptor activator of nuclear factor-κ B ligand
JNK: C-Jun N-terminal kinase
